# Supplementary material for: Immune Enhancement by the Tetra-Peptide Hydrogel as a Promising Adjuvant for an H7N9 Vaccine against Highly Pathogenic H7N9 Virus
Source: Vaccines (Basel). 2022 Jan 17;10(1):130. doi: 10.3390/vaccines10010130 (PMC8778772; doi:10.3390/vaccines10010130)
Supplement: Supplementary file 1 [file vaccines-10-00130-s001.zip › vaccines-1501047-supplementary.pdf]

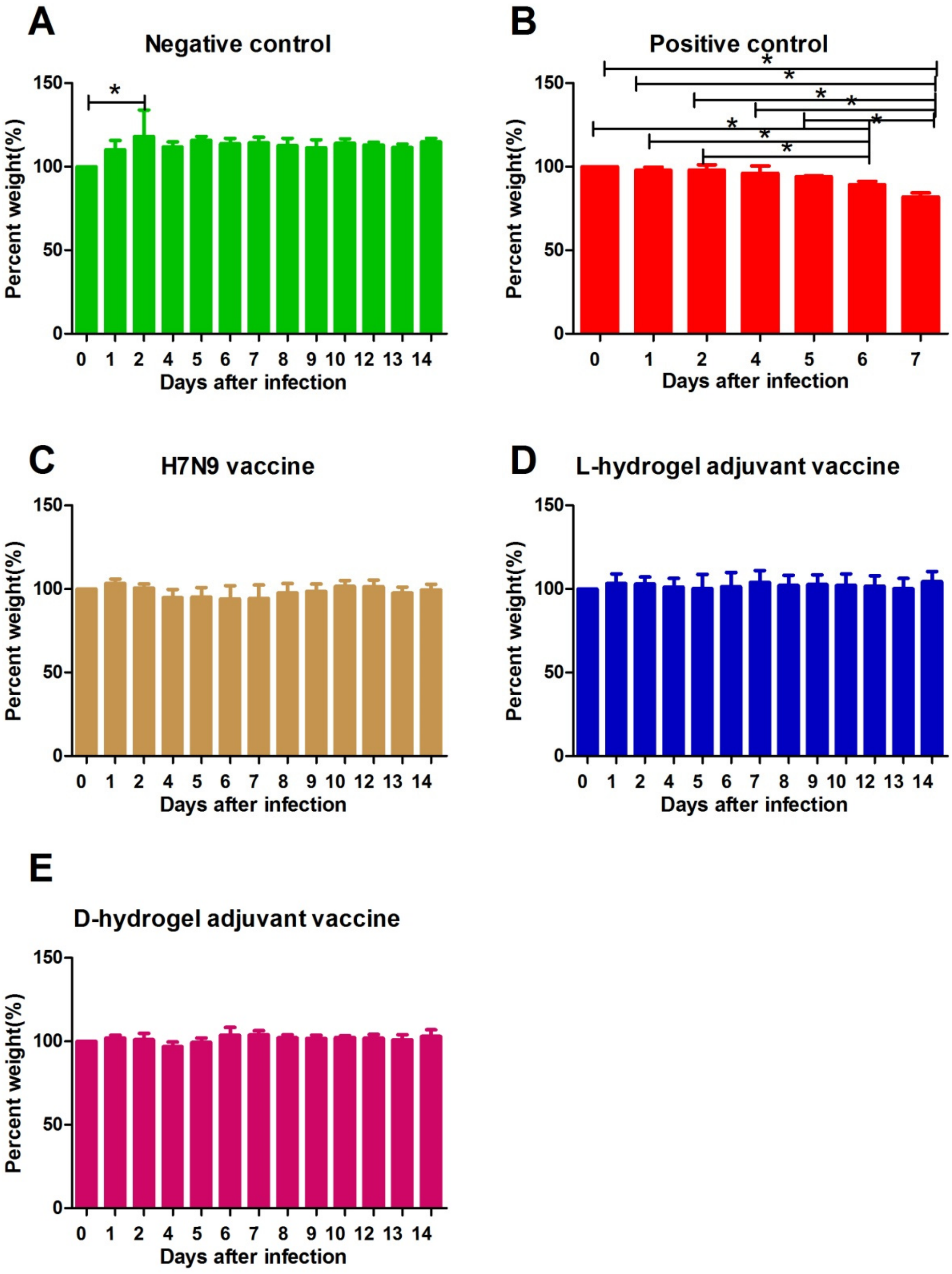

Figure S1. The weight change of mice infected with the H7N9 virus for each group. (A) The weight change of mice in negative control group. (B) The weight change of mice in positive control group. (C) The weight change of mice in H7N9 vaccine group. (D) The weight change of mice in L-Tetra-Peptide hydrogel adjuvant vaccine group. (E) The weight change of mice in D-Tetra-Peptide hydrogel adjuvant vaccine group. The percent weight calculated at 0, 1, 2, 4, 5, 6, 7, 8, 9, 10, 12, 13 and 14 days after infection, respectively. Data are shown as the mean ± SD for the indicated sample sizes. The mean values with error bars representing one standard deviation. An asterisk (∗) represents a p value < 0.05.

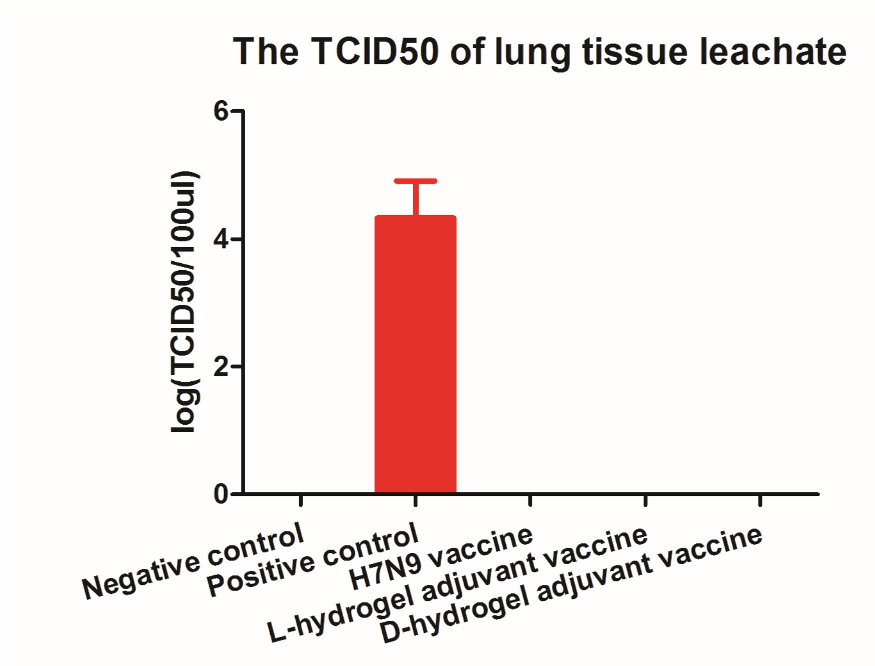

Figure S2. The $TCID_{50}$ of the lung leachate of each group 1 week after virus infection. The tissue leachate was obtained as follows: one milliliter of sterile PBS was added to the frozen tissues in Eppendorf tubes, and then the tissue was cut using sterile scissors. The tubes were centrifuged at 500 x g for 8 min. Then, the supernatant was collected to determine the $TCID_{50}$.
